# BJORL: moving forward, always^☆^

**DOI:** 10.1016/j.bjorl.2018.09.002

**Published:** 2018-09-21

**Authors:** Wilma Terezinha Anselmo-Lima, Shirley Shizue Nagata Pignatari

**Affiliations:** a Universidade de São Paulo (USP), Faculdade de Medicina de Ribeirão Preto (FMRP), Ribeirão Preto, SP, Brazil; b Universidade Federal de São Paulo (UNIFESP), Escola Paulista de Medicina (EPM), Departamento de Otorrinolaringologia e Cirurgia de Cabeça e Pescoço, São Paulo, SP, Brazil

When we decided to write the final editorial for this year's BJORL, we recalled the editorials of 2013 and reviewed what our friends wrote celebrating the journal's 80th birthday. Dr. Sady's editorial^1^ brilliantly, critically and analytically called our attention, to a need for significant changes in the near future to. But, paradoxically, this necessity to take the next steps forward forced us to look back. Simply to look back at an 85-year history, not to perform an inventory of errors. We then went over the previous editorials of our colleagues, because with idealism and competence, without exception, they told us about the trajectory and the challenges that were overcome; this actually constructed the history of our BJORL.^2^, ^3^, ^4^ It was then that we envisioned the scope and challenge of condensing this history into some pages without overlooking names, facts and attitudes. But also to have it guidelines for continuity as proposed by Dr. Sady,^1^ so that years later a colleague would recognize that the most important history is the one he or she is writing at the moment, since it depicts what we are doing today as well as what they did before, before any of us. At the same time we recognize that the greatness of our history has already been documented expressly in the many journal issues that embody 85 years of thinking and practice of our professional colleagues in our specialty in Brazil, leaving us only to humbly compile some facts and authors along this journey. We endorse everything that has been done and reassert everything that has been proposed, is being proposed and will be done from now on. So our work has become simpler.

Where did we come from, what has been done, where are we going? Conceived and founded in 1933 by Drs. Otoni de Rezende and Homero Cordeiro, the Oto-Laryngological Journal of São Paulo began as a printed vehicle of the academic discussions of the Medical Society of São Paulo and later, bearing the name of Brazilian Journal of Otorhinolaryngology, became the vehicle for the publication of specific articles for otorhinolaryngologists all over Brazil. In the 1960s, with the institutionalization of postgraduation courses by the Brazilian Ministry of Education and Culture (MEC), the government agencies that managed these postgraduation courses, such as CAPES and CNPq, started to demand that the resulting studies and researches be published in journals of international scope, that were indexed and had a certain impact factor (IF), so that they were awarded grades in a national ranking and thus could secure public funding for research in the courses and grants for the researchers. This was an essential condition for the promotion of research and, at the same time was a call for a action for the scientific journals that wished to survive in Brazil.^2^, ^3^, ^4^, ^5^, ^6^

Even though these demands were debatable and criticized, their adoption became a milestone for the journal's trajectory. After their implementation there was an improvement in the quality of the journal and the published articles. A result of the outstanding efforts of the Boards that came after that, the journal was gradually indexed by *SciELO*, *Lilacs* and *Experta Medica*. In 2005 we were in *PubMed* and *Medline*, when we started using an English title next to the title in Portuguese. In 2011 we were indexed by the *Institute for Scientific Information (ISI)*. We are currently in the databases for bibliographic consultation of these international indexers.

In parallel to the difficult indexing process, the editors’ perseverance was further impelled by necessary structural changes throughout the entire journal creation process. The main one was the creation of an original publishing platform, that is also currently used by other journals in the country. All issues prior to the journal's indexing have been digitized and are now available for search on the journal's homepage. Among other effects, the advent of informatics and the “wonders” of the internet resulted in an increased number of submissions and in a faster evaluation and approval of articles increasing the journal's exposure abroad. In turn, this last effect brought with it the need for the journal to be published in English, so that it could be distributed *in natura* to the main universities abroad. Thus, in 2009 the Brazilian Journal of Otorhinolaryngology – BJORL (registered in ISSN) was born. Currently we are the most important journal in our specialty in Latin America. As for our presence abroad, it has been well established, and we reached a privileged position that was unthinkable only decades ago. Today our Impact Factor (IF) is 1.412, and only those who know about the difficulties in reaching this small index assigned by ISI (Institute for Scientific Information) are able to measure its importance. We occupy, according to the Journal Citation Reports, the 25th position among the journals of the specialty in the world. We have an ascending history, and that is undeniable.

However, striving to reach the same levels of excellence as the American and European journals is not compatible with complacence. Our responsibility increases as we face the challenges over the next few years, from the changes and transience of government policies to the training/qualification of our specialists in several courses in this continental country. As Dr. Sady^1^ stated in his editorial, we are still far from our ideal. We need to create official mechanisms to consolidate new groups of national researchers with international projection. We need to stimulate scientific production and to encourage basic research in the specialty throughout Brazil. We need to commit the several postgraduate courses to the objectives and standards of BJORL, and the journal, in turn, must also raise its level of stringent requirements for publications aiming at the same level of excellence that is demanded abroad, improving its editorial board, continually training its reviewers, and implementing other tools that foster total quality.

Finally, although we now have other paths and other concerns, we also maintain the same goal as 85 years ago: to make BJORL a reference vehicle, both national and international. History is made, with long strides. It guides us about the starting point, not the point of arrival. The latter will be clarified by the temporal imperatives of the future. Perhaps we are out of our teen years and are now at the age of maturity. Other decades will come. Definitely.

## Conflicts of interest

The authors declare no conflicts of interest.

## References

[1] Costa S.S. (2015). Changing cycles: the necessary rupture to achieve excellence. Braz J Otorhinolaryngol.

[2] Bento R.F. (2013). BJORL – its history until 1996. Braz J Otorhinolaryngol.

[3] Costa H.O. (2013). Read for 80 more years. Braz J Otorhinolaryngol.

[4] de Mello Júnior J.F. (2013). Brazilian Journal of Otorrhinolaryngology – a landmark. Braz J Otorhinolaryngol.

[5] Caldas Neto S. (2013). The soul of octogenarian BJORL. Braz J Otorhinolaryngol.

[6] Crespo A.N., Guimarães A.C. (2013). Otorrhinolaryngology without borders. Braz J Otorhinolaryngol.

